# Evidence of preserved collagen in an Early Jurassic sauropodomorph dinosaur revealed by synchrotron FTIR microspectroscopy

**DOI:** 10.1038/ncomms14220

**Published:** 2017-01-31

**Authors:** Yao-Chang Lee, Cheng-Cheng Chiang, Pei-Yu Huang, Chao-Yu Chung, Timothy D. Huang, Chun-Chieh Wang, Ching-Iue Chen, Rong-Seng Chang, Cheng-Hao Liao, Robert R. Reisz

**Affiliations:** 1 National Synchrotron Radiation Research Center, Hsinchu 30076, Taiwan; 2 Department of Optics and Photonics, National Central University, Chung-Li 32001, Taiwan; 3 Department of Applied Chemistry, National Chiao Tung University, Hsinchu 30010, Taiwan; 4 Dinosaur Evolution Research Center of Jilin University, Changchun, Jilin 130012, China; 5 College of Life Sciences, National Chung Hsing University, Taichung 400, Taiwan; 6 Tosun Public Interests Foundation, Taipei 100, Taiwan; 7 Department of Biology, University of Toronto Mississauga, Mississauga, Ontario, Canada L5L 1C6

## Abstract

Fossilized organic remains are important sources of information because they provide a unique form of biological and evolutionary information, and have the long-term potential for genomic explorations. Here we report evidence of protein preservation in a terrestrial vertebrate found inside the vascular canals of a rib of a 195-million-year-old sauropodomorph dinosaur, where blood vessels and nerves would normally have been present in the living organism. The *in situ* synchrotron radiation-based Fourier transform infrared (SR-FTIR) spectra exhibit the characteristic infrared absorption bands for amide A and B, amide I, II and III of collagen. Aggregated haematite particles (α-Fe_2_O_3_) about 6∼8 μm in diameter are also identified inside the vascular canals using confocal Raman microscopy, where the organic remains were preserved. We propose that these particles likely had a crucial role in the preservation of the proteins, and may be remnants partially contributed from haemoglobin and other iron-rich proteins from the original blood.

## Abstract (web summary)

Biomolecules are rarely preserved during fossilization. Here, Lee and colleagues provide evidence of collagen preserved within the 195-million-year-old rib of a sauropodomorph dinosaur using *in situ* analyses and suggest that haematite particles may have contributed to protein preservation.

Organic biocomponents of animals decompose and degrade during the fossilization process, and minerals in the groundwater are redeposited in the interstitial spaces, slowly transforming vertebrate bones and teeth into inorganic ‘rock'^1^. In some exceptionally preserved fossils, in which the degradation process may have been arrested sufficiently early in the fossilization process, some soft tissue preservation can occur because they are isolated, cemented and protected by inorganic environment and have not been completely permineralized (for example, feathers and claw sheaths). These have always drawn a lot of attention from both the scientific community and the general public not only because of their rarity, but also because of the potential for retrieval of valuable biological and evolutionary information that is generally not available from the permineralized hard skeletons that make up the bulk of fossilized vertebrates^1^. In particular, the preservation of biomolecules associated with cells and blood vessels was uncovered in Cretaceous dinosaurs, and analyses of the chemical composition of these biomaterials were conducted using scanning electron microscopy, transmission electron microscopy, immunological assays and mass spectrometry^2^,^3^. Unfortunately, these studies rarely provided clear and direct *in situ* evidence for entire molecules of protein or collagen preservation without complex demineralization and extraction procedures, leading to the criticism that the signals could be the result of exogenous contaminations, such as bacteria biofilm and haematite (α-Fe_2_O_3_), as shown by using synchrotron-radiation based Fourier transform infrared (SR-FTIR) microspectroscopy and scanning energy dispersive spectroscopy of scanning electron microscopy^4^, respectively. Nevertheless, recent reports have supported the argument that degradation structure of collagen, Gly-Pro-Hyp sequence, was observed using mass spectroscopy and preserved across geological timescales extending 75 million years from the present^5^. This evidence of Gly-Pro-Hyp sequence is matched with the general collagen sequence Gly-X-Y, where the X and Y positions are often proline and hydroxyproline, respectively.

Here we explore the possibility of identifying the organic remains preserved in much older fossil material using *in situ* microspectroscopic methods, rather than by attempting to extract the very small amounts that are likely to be preserved in mid-Mesozoic terrestrial vertebrates. Various skeletal elements of the sauropodomorph dinosaur *Lufengosaurus* from the Lower Jurassic (Early Jurassic, Sinemurian, 190–197 Mya) of Dawa, Lufeng County, Yunnan Province, China, were studied in detail^6^,^7^,^8^; however, surprisingly, it is the rib materials that produced the most significant results (Fig. 1a–m). Adult compact bone (including ribs) is composed of osteons with central vascular canals (Fig. 1b–h) that contain blood vessels and nerves in the living organism and lacunae, or spaces for the adult osteocytes (Fig. 1m).

In order to exclude the possibility of external contamination of the fossil material, in particular from the demineralization process, *in situ* investigation of organic remains within the adult *Lufengosaurus* bones was undertaken using thin sections. FTIR spectroscopy is a state-of-the-art technique and non-destructive analysis method for identifying the vibrational motions of chemical bonding of molecular structures based on the characteristic infrared absorption bands in the mid-infrared range, associated with the various functional groups especially for organic molecules including proteins^9^,^10^,^11^,^12^,^13^. Typically, the organic remains within fossils are rare and would likely be distributed within a tiny space at the micrometre scale. Herein *in situ* SR-FTIR microspectroscopy coupled with ultrahigh brightness infrared synchrotron radiation was utilized not only to provide ultrahigh laterally resolved FTIR spectra but also chemical images related to particular chemical bonding of molecules in a diffraction-limited area to unfold the organic remains within the fossil bone. Raman microscopy is a complementary tool to SR-FTIR microspectroscopy and accessible to provide low vibrational frequencies of chemical bonding of molecules especially for metal oxide compounds in the far-infrared range^14^,^15^, not normally provided by FTIR microspectroscopy. In this investigation dark-field microscopy, synchrotron radiation-based nano-transmission X-ray microscopy (SR-TXM)^16^,^17^ and transient absorption microscopy (TAM)^18^,^19^ were also employed to reveal three-dimensional (3D) lacunae within the fossil bone, and two-dimensional transient absorption images of haematite distributed in the vascular canals and lacunae, respectively. The results indicate that native collagen was preserved within the osteonal central vascular canals, together with haematite particles that were likely derived, at least in part, from haemoglobin of the dinosaur. This suggests that haemoglobin and the resulting haematite may have had a preservative effect on the proteins that originally resided in the central osteonal canals of the rib.

## Results

### SR-FTIR of adult *Lufengosaurus* bone

**Figure 1:**
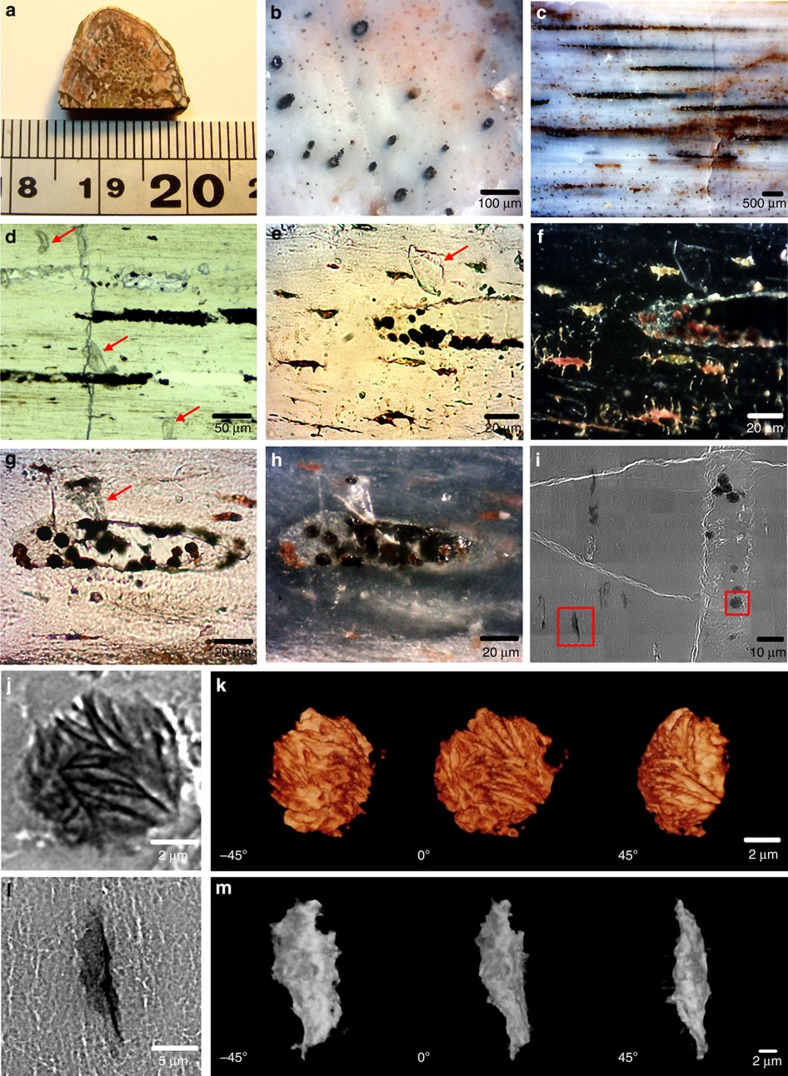
Rib fragment (CXPM Z4644) of *Lufengosaurus*. (**a**) Rib before sectioning, (**b**) transverse section of the rib, small black circles are the central vascular canals in the osteons, (**c**) longitudinal section of the rib showing distribution of infilled vascular canals, (**d**–**h**) close ups of preserved collagen-infilling materials within the vascular canals of the rib; flat transparent preserved protein fragments that were washed out from the cut canals, as explained in the main text, are indicated by red arrows, **f**,**h** are the dark-field images of **e**,**g**, respectively, (**i**) SR-TXM image of microcrystals of haematite within the vascular canal, indicated by red squares, (**j**) microcrystal of haematite inside the vascular canal, (**k**) tomographic images of haematite crystal in different views, (**l**) lacuna within the bone matrix and (**m**) tomographic images of lacunae in different views.

Transversal sections through long bones, including ribs, did not produce a consistent organic signal for in depth investigations; however, there was sufficient evidence to suggest that the central osteonic canals should be the ideal area of the bone to explore for organic remains. Since these vascular canals are longitudinally distributed within the rib, longitudinal thin section slides were made for full accessesment of the materials packed inside these blood vessels and nerve-carrying canals (Fig. 1b,c). Several flat, micron-sized, preserved fragments were found along and inside the vascular canals, together with dark-red micron-sized particles (Fig. 1d–g).

**Figure 2:**
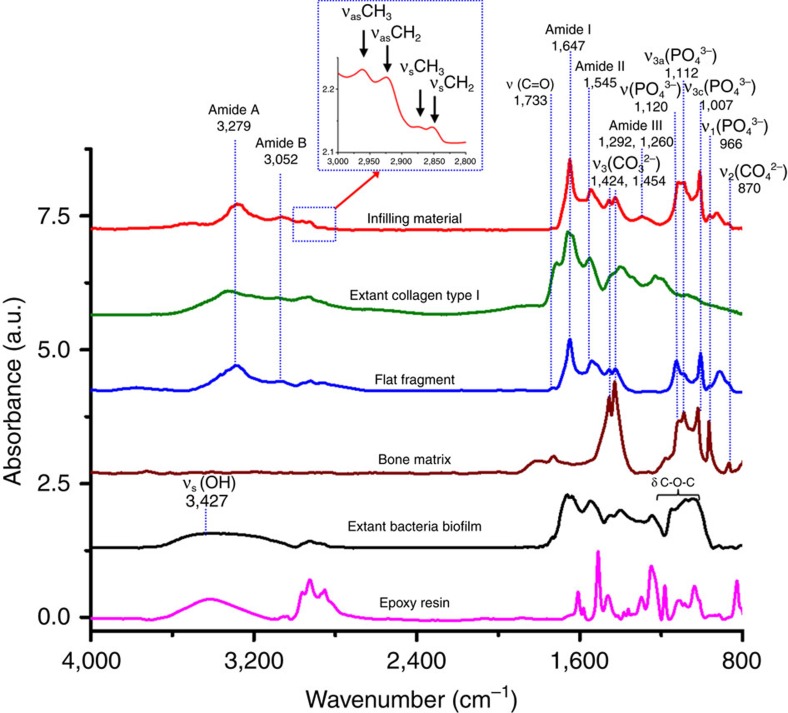
Representative SR-FTIR spectra. Baseline-corrected and normalized characteristic infrared band assignment for preserved collagen within the central vascular canals shown in red, and the peaks were assigned for methyl group (_s_CH_3_ and _as_ CH_3_) and methylene (_s_CH_2_ and _as_ CH_2_) in the spectral range of 3,000–2,800 cm^−1^ as shown in the blue inset, collagen type I from extant calf skin dispersed in 0.1% acetic acid solution in green, preserved protein remains in flat fragments found in and near the central canals of the fossil bone in blue, bone matrix in brown, extant bacteria biofilm in black and epoxy resin in pink. It is evident that the spectra of preserved collagen and extant collagen type I are closely matched. The extant bacterial biofilm showed significant differences from fossil or extant collagen in the range of 3,100–3,600 cm^−1^ region (_s_OH).

Preserved collagen material inside the vascular canals, transparent flat preserved protein fragments, were identified using SR-FTIR (as discussed below), and dark-red particles in both vascular canals and lacunae were also clearly observed by using SR-TXM (Fig. 1i–m). The 3D computed tomographic image of dark-red particles (using SR-TXM) exhibited an aggregate-lamellar crystal structure when attached to the wall of the vascular canals, and a more amorphous structure when found within the lacunae. Infrared absorption bands of the preserved collagen-infilling material within the central vascular canals were observed at 3,279, 3,052, 1,649, 1,637, 1,545, 1,292 and 1,260 cm^−1^ by using SR-FTIR microspectroscopy (Fig. 2), which were consistent with the characteristic infrared absorption bands of collagen type I and elastin^20^,^21^,^22^,^23^,^24^, and assigned to amide A band (-N-H stretching vibration of peptide bond of protein), amide B band (overtone of amide II band of peptide bond of protein), amide I band (carbonyl group, >C=O, stretching vibration of peptide bond of protein, lower vibration wavenumbers being due to hyperconjugation of peptide side chain of protein as compared with other carbonyl compounds), characteristic absorption for triple helix of collagen type I, amide II band (a coupling vibration of C-N stretching vibration and C-N-H bending vibration of peptide bond of protein) and amide III band (C-N stretching vibration and N-H deformation absorption of collagen and elastin), respectively (Supplementary Table 1).

SR-FTIR spectra of the transparent flat protein fragments on the bone surface were similar to that of the preserved collagen-infilling material inside the vascular canals, with weak amide III bands at 1,292 and 1,260 cm^−1^ (Fig. 2). These infrared absorption bands of protein material were also matched with extant collagen type I extracted from the skin of a modern living animal (calf), except for the intense infrared absorption band at 1,715 cm^−1^ of the extant collagen type I being attributed to the carbonyl group (>C=O) of acetic acid (HAc) utilized to disperse collagen in solution by using 0.1% solution in 0.1 M HAc (Fig. 2). Thus, a combination of transparent flat preserved protein fragments was found inside the vascular canals as well as near the canals, adhered on the bone surface (Fig. 1d–h). The protein remains within the rib were mixed with inorganic bone component carbonate and phosphate (Fig. 2).

The characteristic broad infrared absorption band of hydroxyl group (-OH) of polysaccharides was not observed in the range of 3,700–3,100 cm^−1^, normally attributed to the absorption of the cell wall of bacteria^25^ as in the extant bacterial biofilm of *Saccharomyces cerevisiae* (Fig. 2). Hundreds of strains for filamentous fungi were investigated using FTIR spectroscopy and partial least squares discriminant analysis^26^,^27^, and these showed a greater infrared absorption band of hydroxyl groups (-OH) and glycosidic bonds (-C-O-C-) of polysaccharides, the dominant component of bacterial cell wall. Furthermore, the FTIR spectra of infilling material and flat fragments distributed along and around the central vascular canals within the fossil showed none of characteristic infrared absorption of epoxy resin remains as compared with the spectrum of epoxy resin used here (Fig. 2).

On the basis of the fundamental structure of collagen, left-handed triple helix is the main character of collagen type I, which is made of the repetitious amino-acid sequence and Glycine-X-Y is the most abundant, in which X and Y are frequently proline or hydroxyproline, respectively, for both the blood vessel wall and bone matrix protein. The SR-FTIR spectrum of preserved collagen-infilling material presented the characteristic infrared absorption for left-handed triple helix structure of collagen remains at 1,637 cm^−1^ in amide I band, non-polar triple helix of collagen at 1,292 cm^−1^ and polar triple helix of collagen and elastin at 1,260 cm^−1^ in amide III band in amide III band for collagen type I (refs 23, 28, 29, 30). Specifically, we also found the presence of a deconvoluted band at 1,337 cm^−1^, which we propose to be attributable predominantly to the CH_2_-wagging vibration of proline side chains of glycine-X-Y sequence structure of collagen^22^ (Supplementary Fig. 2 and Supplementary Table 2).

These spectral findings provide strong evidence that the preserved collagen-infilling material within the vascular canals of the rib of *Lufengosaurus* is the preserved ancient native collagen type I, consistent with the characteristic infrared absorptions of protein^31^ and collagen^32^,^33^,^34^. We propose that the carbonated apatite (CAP) of the bone matrix likely played a crucial protection role as the cementing material for the protein and collagen remains within the vascular canals for reducing the possibility of further degradation.

The results of the FTIR spectra of the bone matrix exhibited an infrared absorption doublet band of carbonate (ν_3_ CO_3_^2−^) at 1,454 and 1,424 cm^−1^, an infrared absorption band of carbonate (ν_2_ CO_3_^2−^) at 870 cm^−1^, and four infrared absorption bands of phosphate (ν PO_4_^3−^, ν_3a_ PO_4_^3−^, ν_3c_ PO_4_^3−^ and ν_1_ PO_4_^3−^) at 1,120, 1,088, 1,007 and 966 cm^−1^, in agreement with several studies^35^,^36^,^37^,^38^,^39^ (Supplementary Table 1). SR-FTIR spectra of the bone matrix showed that most of the hydroxyl groups of apatite were absent, and instead, contained many carbonate and phosphate substitutions. Furthermore, a considerable broadening of the 950–1,200 cm^−1^ phosphate bands was observed as the Mg content increased, also in agreement with Zyman *et al*.^40^ In a sense, chemically stable CAP appears to have ‘cemented/entombed' the organic remains isolated within the vascular canals, and SR-FTIR spectral images of the bone showed that CAP surrounded the central vascular canal (Supplementary Fig. 3).

In light of these findings, we argue that a small amount of endogenous collagen and protein were isolated and preserved in the tubular space of the vascular canals of the rib osteons. Collagen and protein are hydrophilic materials; therefore, some collagen and protein fragments that were originally located within the canals were washed out of the vascular canals by the distilled water during the early sample polishing procedure of bone fossil slide (Supplementary Fig. 3a), and were therefore found on the surface.

### Raman spectroscopy of adult *Lufengosaurus* bone

**Figure 3:**
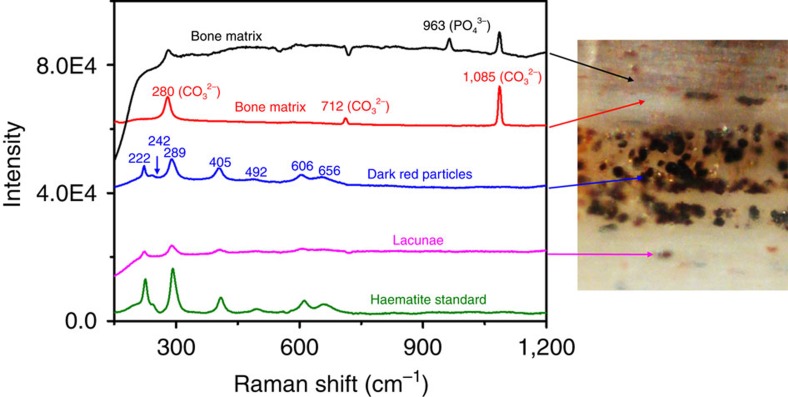
Comparative Raman spectra. Black and red lines represent the bone matrix around and near the vascular canal; blue and magenta lines represent the dark-red microparticles located in the vascular canal and in the lacunae, respectively. The dark-red particles' spectrum closely matches that of the haematite standard (green line, bottom), indicating that these dark-red microparticles are haematite. The spectra (black and red) from the areas outside the vascular canal indicate that there is no haematite in the bone's apatite matrix.

The dark-red particles found within the central vascular canals were analysed by using confocal Raman microscopy. Raman scattering peaks of dark-red micron-sized particles were observed at 222 cm^−1^ (A_1g_ (1)), 242 cm^−1^ (E_g_ (1)), 289 cm^−1^ (E_g_ (2)), 296 cm^−1^ (E_g_ (3)), 405 cm^−1^ (E_g_ (4)), 492 cm^−1^ (A_1g_ (2)), 606 cm^−1^ (E_g_ (5)) and 656 cm^−1^ (E_u_ (LO)). These observed Raman peaks were assigned to haematite (α-Fe_2_O_3_), which was consistent with the Raman scattering signals of standard haematite and previous studies^41^,^42^ (Fig. 3). Surprisingly, haematite was also observed within the lacunae, where the osteocytes resided, even though the connecting networks by canaliculi among lacunae connected to the vascular canals were blocked by carbonate infills.

**Figure 4:**
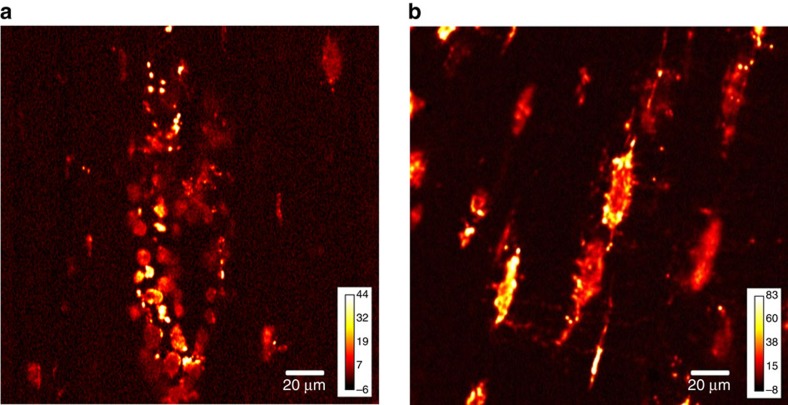
Transient absorption microscopy of *Lufengosaurus* rib. Transient absorption images of vascular canal (**a**) and lacunae (**b**). These show high concentration of haematite particles.

The TAM images of α-Fe_2_O_3_ within the fossil section also demonstrated that haematite particles were localized within the vascular canals and the lacunae (Fig. 4), but were rarely found within the concentric osteonic bone tissues or the encasing sediment. We propose that small chambers were formed by concretions of haematite and carbonate within the central vascular canals, preserving isolated collagen and protein remains (Fig. 1f-h).

Osteocytes housed within lacunae are affected and proven to stimulate osteoblast proliferation by lactoferrin, a kind of iron glycoproteins, considered to be a potent anabolic factor, and lactoferrin is also one of the transferrin proteins that play a key role for transferring iron ion into the osteocytes and regulating the level of free iron ion in the blood and in external secretions. The affinity of lactoferrin for iron ion is demonstrated to be 300 times higher than that of other transferrin^43^,^44^, and lactoferrin has the ability to promote bone formation and inhibit bone resorption^45^,^46^. We therefore propose that iron ions bound by lactoferrin or other iron-rich transferrin proteins may be abundantly distributed within the osteocytes in the micron-scale housing of lacunae during the lifetime of a dinosaur and even after fossilization (Fig. 4b).

Thermodynamically stable haematite within the fossil rib may be the final product after an extremely long period of oxidation reaction, contributed from the degradation of iron-containing haemoglobin, myoglobin, lactoferrin and other iron-rich proteins that normally existed in the circulatory network of osteocytes. Goethite (α-FeOOH, ferric hydroxide), quartz and kaolin clay are the major components of the encasing sediment matrix around the fossil, and this identification is based on the Raman signals of Si–O band and Al–O–Si band at 463 and 658 cm^−1^, respectively, at the fossil site^47^ (Supplementary Fig. 4); however, neither characteristic Raman signals of quartz and kaolinite was found in the vascular canals or the lacunae.

It is possible that the vascular canals could have provided conduits for transporting iron ions in the groundwater into the bone postmortem, but the source of iron ion (Fe^3+^) should be mainly from the goethite dissolved in the groundwater around the bone fossil. However, goethite is sparingly soluble in neutral pH water and the concentration of iron ion (Fe^3+^) is ∼10^−16.9^–10^−11.9^ M l^−1^ (10^6.1^–10^12.1^ ions per litre) from goethite, with the solubility constant *K*_sp_ being ∼10^−41^ and 10^−43^ for goethite and haematite in the neutral water solution^48^,^49^, respectively. Therefore, a single 10-μm spherical particle of haematite requires at least 2.1 × 10^13^ iron ions and needs about 20 l groundwater to traverse the tiny channel of the vascular canal. Besides, formation of goethite is favoured at low pH values (pH 2–5) and high values (pH 10–14), while that for haematite is favoured at around neutral pH values^50^. The activation energy of transformation from goethite to haematite requires 169±8 and 154±15 kJ mol^−1^ for an ore mineral and a recent sedimentary goethite, respectively, based on the results of thermogravimetric, transmission electron microscopy and X-ray diffraction^51^. In addition, the phase transformation for goethite to haematite occurs at ∼250 °C in vacuum as shown using X-ray power diffraction^52^. Iron (III) oxide–hydroxide is the main source of goethite and haematite, which can turn into goethite and haematite under different pH conditions near room temperature. The ratio of goethite/haematite in soils was observed to increase with a decreasing pH from 0.4 to 5.6 within the soil^53^. Furthermore, goethite was formed only at pH 12, and maximum haematite formation, ∼71% haematite, occurred at a pH range of 7–8. Mixtures of goethite and haematite were formed at all other pH values^54^, and there was little spectral evidence of goethite being present in the vascular canals of the fossil. We therefore propose that the possibility for goethite dehydration and then direct transformation to haematite within the rib of the sauropodomorph *Lufengosaurus* is extremely low.

Ferrihydrite, 2-line and 6-line ferrihydrite (5Fe_2_O_3_·9H_2_O), can easily transform to goethite or haematite, and the solubility constant of ferrihydrite (*K*_sp_=10^−39^) is higher than that of goethite and haematite in groundwater^47^,^48^,^49^. Ferrihydrite could be a possible precursor of haematite found within the fossil bones due to its low thermodynamic stability. Interestingly, ferrihydrite frequently plays a key role in intracellular iron storage of the ferritin protein in many living organisms, and decaying organics may trigger the haematite concretion in the vascular canals^55^,^56^,^57^. Thus, the likelihood that the source of the haematite particles is from endogenous iron-rich proteins, including haemoglobin that circulated within the vascular canals, is high.

As already discussed above, small segments of the central vascular canals appear to have been isolated by haematite and carbonate concretions, preventing infiltration of outside material, and preventing complete degradation of organic remains (Fig. 1b–d). Thus, collagen and protein remains were kept in the vascular canals^55^,^56^,^57^, and we suggest that the aggregated dark-red micron-sized particles of haematite played a key role in the preservation of organic material.

## Discussion

We report the presence of ancient collagen and protein remains preserved in a 195 million-year-old fossil, as demonstrated through *in situ* SR-FTIR microspectroscopy of the Early Jurassic sauropodomorph dinosaur *Lufengosaurus*. Previously, only some evidence of preservation of organic remains was found in embryonic fossils of the same Early Jurassic sauropodomorph dinosaur *Lufengosaurus* from the same locality in Yunnan Province, China. In addition, the next oldest degraded collagen fragments were found in 75 million-year-old fossils^5^,^8^. The characteristic infrared absorption spectra of collagen and protein provide undeniable, clear evidence that collagen and protein remains were preserved inside the osteonal central vascular canals of this early dinosaur. This finding extends the record of preserved organic remains more than 100 million years, and highlights the importance of using *in situ* approaches to these types of investigations.

We propose that haematite cementation may have played an important role in the preservation of these organic remains, isolated within the vascular canals by haemoglobin-derived haematite aggregations. The concentration of molecules of haemoglobin in each erythrocyte is very high. For example, there are ∼280 million molecules of haemoglobin in a single erythrocyte of human blood^58^, yielding a total content of 1.12 billion iron ions binding to molecules of haemoglobin in a single erythrocyte. Therefore, the number of iron ions of a single 10 μm spherical particle of haematite is approximately equal to the number of iron ions contributed from 20,000 red blood cells, according to the molecular volume 30.47 cm^3^ mol^−1^ of haematite^59^,^60^.

Normally, tissues of terrestrial vertebrates degrade quickly after death. The time period for degradation of organic materials within bones is thought to be shorter than that of bone permineralization, and, therefore, haematite concretions need to form relatively early in order to prevent complete degradation of protein materials within the osteonal vascular canals.

It is therefore likely that the main iron ion source to trigger the haematite concretion formation is endogenous. The aggregation of exogenous iron is expected to take a much longer time to form haematite than that of endogenous iron source, at least partly because of the extremely low solubility of goethite and haematite in groundwater. Nevertheless, it is possible that the observed haematite could have also had some exogenous component.

If our interpretation is correct, then the preservation of endogenous collagen and protein by iron ions was attributable to the presence of iron-containing haemoglobin of red blood cells and lactoferrin, these having played a key role as antioxidants for preventing further oxidation of collagen and protein in the blood vessel distributed within the osteons of the compact bone of this fossil. The ferrous ion bound to haeme chromophores and lactoferrin may have also played a role as electron donors for free-radical-medicated fixation and antimicrobial activity, and the active sites of protein might be blocked by iron ions for preventing further degradation^61^,^62^,^63^,^64^,^65^. Iron ions circulated in living bone not only inside the bone matrix through blood vessels in the osteonal vascular canals, but also to bone osteocytes through the lacunocanalicular network^66^,^67^. We therefore propose that the iron ions would be mainly localized within the vascular canals and the lacunae within the bones, as we see it occurring in the rib, and collagen in the canal was isolated from the outside environment and surrounded by haematite concretion and CAP, like a closed micro-sized chamber of vascular space. Some isolated collagen would be prevented from oxidation during the redox reaction at the time of fossilization. Interestingly, ∼90% of the bone matrix proteins is collagen in extant species^68^; however, little or no protein or collagen was found within the bone matrix of the *Lufengosaurus* rib, as indicated by the absence of clear characteristic infrared absorption of collagen or endogenous protein in SR-FTIR microspectroscopy. Therefore, we suggest that the preserved collagen type I may be derived from the remains of the blood vessels that resided in the osteonal canals and mixed with iron-related materials derived from haemoglobin of red blood cells and lactoferrin, with some possible additional contribution from the iron ion-carrying groundwater.

Our study has shown that SR-FTIR microspectroscopy and confocal Raman spectroscopy provide crucial evidence for the preservation of protein/collagen as compositional constituents of fossils. This *in situ* analysis of 195-million-year-old fossil material from the early stages of dinosaur evolution, without any decalcification processes or other chemical treatment, has yielded significant results while also eliminating the potential of introducing other organic contaminations or alter the chemical components. Finally, future enhanced methods are likely to lead to identification of the degradation components of collagen type I, and other organic remains across greater geologic timescales than previously considered possible.

## Methods

### Tissue preparation

Skeletal elements of an adult *Lufengosaurus* were collected and studied (specimens housed in the ChuXiong Prefectural Museum, catalogue CXPM Z4644). Initially, there was no evidence of soft tissue preservation in transversely sectioned samples. However, longitudinally sectioned rib samples, especially those that exposed the osteonal central vascular canals, revealed the presence of several interesting, informative, transparent flat fragments, as well as numerous dark-red aggregated microparticles (Fig. 1b–h and Supplementary Fig. 1).

In order to exclude external organic and chemical contaminations during the preparation of the fossil sample slides, the bone samples were not demineralized by chemical treatments. Instead, the fossil sample was cut to a thickness of 1 mm and glued on a glass slide with epoxy resin. The sample was then ground down using 30, 15, 6, 3, 1 and 0.5 μm diamond-lapping film (661X, 3M, USA), with distilled water on a rotary polishing machine (Metprep3, Allied, USA) to produce slides of ∼30 μm in thickness. The resulting fossil material was separated from the epoxy glue, and was polished by hand with gloves and careful handling until the sample thickness was reduced to ∼10 μm. During the final stages of the polishing procedure, deionized (DI) water was replaced with ethanol (95% *v*/*v*; XR-LETOH-B, Uni-Onward corp., New Taipei City, Taiwan) in order to prevent proteins that were probably aggregated within vascular canals from being dissolved or dispersed by attractive electrostatic and dipole forces of DI water. It is also important to note that in all samples used in this study the epoxy resin, originally used to mount the samples on glass slides, was carefully and completely removed with acetone (AS-1112, TEDIA, Fairfield, OH, USA) and the fossil sample was transferred on Ag/ SnO_2_-coated infrared reflective low-e slides (Kevley Technologies, Chesterfield, OH, USA) for the SR-FTIR microspectroscopic scan.

### SR-FTIR microspectroscopy

In this investigation, the *in situ* preserved collagen was measured directly by using SR-FTIR microspectroscopy at the beam line BL14A1 infrared microspectroscopy (IMS) endstation of the National Synchrotron Radiation Research Center (NSRRC)^69^, which includes a FTIR spectrometer (Nicolet 6700, ThermoFisher Scientific, Madison, WI, USA) and a confocal infrared microscope (Nicolet Continuum; ThermoFisher Scientific, Madison, WI, USA). The highly collimated synchrotron infrared beam was directed into the IMS endstation, and focused to a 10 × 10 μm^2^ infrared spot by a 32 × Cassegrain objective on the ultrathin slides loaded into the infrared confocal microscope. This procedure, required in order to acquire high signal/noise ratio FTIR spectra of the fossil samples, was preferred over the larger 50 × 30 μm^2^ infrared spot, and was achieved by using a conventional global infrared source^70^. The SR-FTIR spectra were collected in the mid-infrared range of 4,000–650 cm^−1^, at spectral resolution of 4 cm^−1^, with a total of 128 scans at confocal aperture of 10 × 10 μm^2^, with lateral mapping step size of 10 μm. The optical path of the IMS endstation of BL14A1 was continuously purged by using dry nitrogen evaporated from liquid nitrogen in Dewar (XL-100, Taylor-Wharton, Theodore, AL, USA) to replace most of carbon dioxide and water vapour in the optical path. In addition, an automatic atmospheric suppression function in OMNIC (OMNIC 9.2, 2012; ThermoFisher Scientific Inc., Waltham, MA, USA) was employed for eliminating rovibration absorptions of carbon dioxide and water vapour in the ambient air.

### Other analyses and imaging

Micro-Raman spectra of the preserved collagen materials in the central vascular canals and the bone matrix were also acquired by using a home-built confocal Raman microscope, equipped with a monochromator (Shamrock SR 303i-A, Andor Technology, USA), He-Ne laser (25-LHP-928-249, CVI Melles Griot, USA), a thermo-electric cooling CCD (DU 401-BR-DD-968, Andor, USA) and a microscope (BX51, Olympus, Tokyo, Japan). The haematite particles were imaged and studied using the SR-TXM at BL01B1 of NSRRC for full-field nano-imaging^16^,^17^ and TAM at the Department of Applied Chemistry of National Chiao Tung University for full-field absorption imaging^18^,^19^, respectively.

### Data availability

The data set generated and analysed during the current study is available from the lead author on reasonable request.

## Additional information

**How to cite this article:** Lee, Y.-C. *et al*. Evidence of preserved collagen in an Early Jurassic sauropodomorph dinosaur revealed by synchrotron FTIR microspectroscopy. *Nat. Commun.* **8,** 14220 doi: 10.1038/ncomms14220 (2017).

**Publisher's note:** Springer Nature remains neutral with regard to jurisdictional claims in published maps and institutional affiliations.

## Supplementary Material

Supplementary Information Supplementary Figures, Supplementary Tables and Supplementary References

## References

[b1] Schweitzer M. H. Soft tissue preservation in terrestrial Mesozoic vertebrates. Annu. Rev. Earth Planet. Sci. 39, 187–216 (2011).

[b2] Schweitzer M. H. . Analyses of soft tissue from *Tyrannosaurus rex* suggest the presence of orotein. Science 316, 277–280 (2007). 17431179 10.1126/science.1138709

[b3] Schweitzer M. H., Wittmeyer J. L. & Horner J. R. Soft tissue and cellular preservation in vertebrate skeletal elements from the Cretaceous to the present. Proc. R. Soc. B 274, 183–197 (2007). 10.1098/rspb.2006.3705 PMC1685849 17148248

[b4] Asara J. M., Schweitzer M. H., Freimark L. M., Phillips M. & Cantley L. C. Protein sequences from mastodon and *Tyrannosaurus rex* revealed by mass spectrometry. Science 316, 280 (2007). 17431180 10.1126/science.1137614

[b5] Bertazzo S. . Fibres and cellular structures preserved in 75-million-year-old dinosaur specimens. Nat. Commun. 6, 7352 (2015). 26056764 10.1038/ncomms8352 PMC4468865

[b6] Bien M. N. ‘‘Red Beds'' of Yunnan. Bull. Geol. Soc. China 21, 159 (1941).

[b7] Fang X. . in Proceedings of the Third National Stratigraphical Congress of China, 208–214 (Geological Publishing House, Beijing, China, 2000).

[b8] Reisz R. R. . Embryology of Early Jurassic dinosaur from China with evidence of preserved organic remains. Nature 496, 210–214 (2013). 23579680 10.1038/nature11978

[b9] Lebon M. . Imaging fossil bone alterations at the microscale by SR-FTIR microspectroscopy. J. Anal. At. Spectrom. 26, 922–929 (2011).

[b10] Brink K. S. . Developmental and evolutionary novelty in the serrated teeth of theropod dinosaurs. Sci. Rep. 5, 12338 (2015). 26216577 10.1038/srep12338 PMC4648475

[b11] Surmik D. . Spectroscopic studies on organic matter from Triassic reptile bones, Upper Silesia, Poland. PLoS ONE 11, e0151143 (2016). 26977600 10.1371/journal.pone.0151143 PMC4792425

[b12] Lang L., Kirsimäe K. & Vahur S. Diagenetic fate of bioapatite in linguliform brachiopods: multiple apatite phases in shells of Cambrian lingulate brachiopod *Ungula ingrica* (Eichwald). Lethaia 49, 13–27 (2016).

[b13] Jadwiszczak P. . The first record of fossil penguins from East Antarctica. Antarct. Sci. 25, 397–408 (2013).

[b14] Thomas D. B., Fordyce R. E., Frew R. D. & Gordon K. C. A rapid, non-destructive method of detecting diagenetic alteration in fossil bone using Raman spectroscopy. J. Raman Spectrosc. 38, 1533–1537 (2007).

[b15] Schopf J. W. & Kudryavtsev A. B. Confocal laser scanning microscopy and Raman imagery of ancient microscopic fossils. Precambrian Res. 173, 39–49 (2009).

[b16] Yin G.-C. . Sub-30 nm Resolution X-ray Imaging at 8 keV using third order diffraction of a zone plate lens objective in a transmission microscope. Appl. Phys. Lett. 88, 241115 (2006).

[b17] Wang C.-C. . Evolution and function of dinosaur teeth at ultra-microstructural level revealed using synchrotron transmission X-ray microscopy. Sci. Rep. 5, 15202 (2015). 26512629 10.1038/srep15202 PMC4625602

[b18] Jung Y. . Fast detection of the metallic state of individual single-walled carbon nanotubes using a transient-absorption optical microscope. Phys. Rev. Lett. 105, 217401 (2010). 21231351 10.1103/PhysRevLett.105.217401

[b19] Villafana T. E., Brown W., Warren W. S. & Fischer M. Ultrafast pump-probe dynamics of iron oxide based earth pigments for applications to ancient pottery manufacture. In *Proc. SPIE 9527, Optics for Arts, Architecture, and Archaeology V*, 952709 (SPIE, Munich, Germany, 2015).

[b20] Cooper E. A. & Knutson K. in *Physical Methods to Characterize Pharmaceutical Proteins* (eds Herron, J. N., Jiskoot, W. & Crommelin, D. J. A.), 101-143, (Plenum, 1995).

[b21] Wetzel D. L., Post G. R. & Lodder R. A. Synchrotron infrared microspectroscopic analysis of collagens I, III, and elastin on the shoulders of human thin-cap fibroatheromas. Vib. Spectrosc. 38, 53–59 (2005).

[b22] Jackson M., Choo L. P., Watson P. H., Halliday W. C. & Mantsch H. H. Beware of connective tissue proteins: assignment and implications of collagen absorptions in infrared spectra of human tissues. Biochim. Biophys. Acta 1270, 1–6 (1995). 7827129 10.1016/0925-4439(94)00056-v

[b23] Vedantham G. . A holistic approach for protein secondary structure estimation from infrared spectra in H_2_O solutions. Anal. Biochem. 285, 33–49 (2000). 10998261 10.1006/abio.2000.4744

[b24] Karima B., Razia N., Gilles G. & Cyril P. Collagen types analysis and differentiation by FTIR spectroscopy. Anal. Bioanal. Chem. 395, 829–837 (2009). 19685340 10.1007/s00216-009-3019-y

[b25] Kaye T. G., Gaugler G. & Sawlowicz Z. Dinosaurian soft tissues interpreted as bacterial biofilms. PLoS ONE 3, e2808 (2008). 18665236 10.1371/journal.pone.0002808 PMC2483347

[b26] Lecellier A. . Implementation of an FTIR spectral library of 486 filamentous fungi strains for rapid identification of molds. Food Microbiol. 45, 126–134 (2015). 25481069 10.1016/j.fm.2014.01.002

[b27] Lecellier A. . Differentiation and identification of filamentous fungi by high-throughput FTIR spectroscopic analysis of mycelia. Int. J. Food Microbiol. 168-169, 32–41 (2014). 24231128 10.1016/j.ijfoodmicro.2013.10.011

[b28] Krimm S. & Bandekar J. Vibrational spectroscopy and conformation of peptides, polypeptides, and proteins. J. Adv. Protein Chem. 38, 181–364 (1986). 10.1016/s0065-3233(08)60528-8 3541539

[b29] Cooper E. A. & Knutson K. in *Physical Methods to characterize Pharmaceutical Proteins. Volume 7 of the series Pharmaceutical Biotechnology: Fourier Transform Infrared Spectroscopy Investigations of Protein Structure* (eds Herron, J. N. Jiskoot, W. & Crommelin, D. J. A.), 101–143 (Plenum, 1995). 10.1007/978-1-4899-1079-0_3 8564016

[b30] Schliephake H. & Scharnweber D. Chemical and biological functionalization of titanium for dental implants. J. Mater. Chem. 18, 2404–2414 (2008).

[b31] Barth A. & Haris P. I. Biological and Biomedical Infrared Spectroscopy IOS Press, STM Publishing House (2009).

[b32] Alejandro H. . Thermal, infrared spectroscopy and molecular modeling characterization of bone: an insight in the apatite-collagen type I interaction. Adv. Biol. Chem. 3, 215–223 (2013).

[b33] Nagai1 T., Suzuki N., Tanoue Y. & Kai N. Collagen from tendon of Yezo Sika Deer (*Cervus nippon* *yesoensis*) as by-product. Food Nutr. Sci. 3, 72–79 (2012).

[b34] Figueiredo M. M., Gamelas J. A. F. & Martins A. G. in *Infrared Spectroscopy - Life and Biomedical Sciences* (ed. Theophanides, T.) Ch. 18 (InTech, 2012).

[b35] Marshall A. O. & Marshall C. P. Vibrational spectroscopy of fossils. Palaeontology 58, 201–211 (2015).

[b36] Capanema N. S. V. . Niobium-doped hydroxyapatite bioceramics: synthesis, characterization and *in vitro* cytocompatibility. Materials 8, 4191–4209 (2015). 10.3390/ma8074191 PMC5455653 28793433

[b37] Garskaite E. . Effect of processing conditions on the crystallinity and structure of carbonated calcium hydroxyapatite (CHAp). Cryst. Eng. Commun. 16, 3950–3959 (2014).

[b38] Pena J. & Vallet-Regi M. Hydroxyapatite, tricalcium phosphate and biphasic materials prepared by a liquid mix technique. J. Eur. Ceram. Soc. 23, 1687–1696 (2003).

[b39] Cacciotti I., Bianco A., Lombardi M. & Montanaro L. Mg-substituted hydroxyapatite nanopowders: synthesis, thermal stability and sintering behavior. J. Eur. Ceram. Soc. 29, 2969–2978 (2009).

[b40] Zyman Z. . Magnesium-substituted hydroxyapatite ceramics. Materwiss Werksttech 37, 474–477 (2006).

[b41] Chaudhari N. S. . Maghemite (hematite) core (shell) nanorods via thermolysis of a molecular solid of Fe-complex. Dalton Trans. 40, 8003–8011 (2011). 21720640 10.1039/c1dt10319a

[b42] Shim S.-H. & Duffy T. S. Raman spectroscopy of Fe_2_O_3_ to 62 GPa. Am. Miner. 87, 318–326 (2001).

[b43] Lorget F. . Lactoferrin reduces *in vitro* osteoclast differentiation and resorbing activity. Biochem. Biophys. Res. Commun. 296, 261–266 (2002). 12163011 10.1016/s0006-291x(02)00849-5

[b44] Mazurier J. & Spik G. Comparative study of the iron-binding properties of human transferrins. I. Complete and sequential iron saturation and desaturation of the lactotransferrin. Biochim. Biophys. Acta 629, 399–408 (1980). 6770907 10.1016/0304-4165(80)90112-9

[b45] Cornish J. Lactoferrin promotes bone growth. Biometals 17, 331–335 (2004). 15222486 10.1023/b:biom.0000027713.18694.91

[b46] Naot D., Grey A., Reid I. R. & Cornish J. Lactoferrin – a novel bone growth factor. Clin. Med. Res. 3, 93–101 (2005). 16012127 10.3121/cmr.3.2.93 PMC1183439

[b47] Bikiaris D. . Ochre-differentiation through micro-Raman and micro-FTIR spectroscopies: application on wall paintings at Meteora and Mount Athos, Greece. Spectrochim. Acta A Mol. Biomol. Spectrosc. 56, 3–18 (1999). 10.1016/s1386-1425(99)00134-1 10728852

[b48] Schwertmann U. Solubility and dissolution of iron oxides. Plant Soil 130, 1–25 (1991).

[b49] Hansel C. M., Shawn G. B., Nico P. & Fendorf S. Structural constraints of ferric (hydr)oxides on dissimilatory iron reduction and the fate of Fe(II). Geochim. Cosmochim. Acta 68, 3217 (2004).

[b50] Cudennec Y. & Lecerf A. The transformation of ferrihydrite into goethite or hematite, revisited. J. Solid State Chem. 179, 716–722 (2006).

[b51] Goss C. J. The kinetics and reaction mechanism of the goethite to hematite transformation. Mineralol. Mag. 51, 437–451 (1987).

[b52] Gualtieri A. F. & Venturelli P. *In situ* study of the goethite-hematite phase transformation by real time synchrotron powder diffraction. Am. Mineral. 84, 895–904 (1999).

[b53] Schwertmann U. & Murad E. Effect of pH on the formation of goethite and hematite from ferrihydrite. Clays Clay Miner. 31, 277–284 (1983).

[b54] Kampf N. & Schwertmann U. Goethite and hematite in a climosequence in southern Brazil and their application in classification of kaolinitic soils. Geoderma 29, 27–39 (1983).

[b55] Chasteen N. D. & Harrison P. M. Mineralization in ferritin: an efficient means of iron storage. J. Struct. Biol. 126, 182–194 (1999). 10441528 10.1006/jsbi.1999.4118

[b56] Lewin A., Moore G. R. & Brun N. E. L. Formation of protein-coated iron minerals. Dalton Trans. 22, 3597–3610 (2005). 10.1039/b506071k 16258608

[b57] Russell D. A., Fisher P. E., Barrick R. E. & Stoskopf M. K. Response: dinosaur with a heart of stone. Science 291, 783a (2001).11157158

[b58] Woodhouse A. Revise AS Biology for OCR Heinemann Educational Publishers (2001).

[b59] Sahebary M., Raygan S., Ebrahimi S. A. S. & Abdizadeh H. Inception of transformation of hematite to magnetite during mechanical activation: a thermodynamical approach. IJSTC 33, 415–424 (2009).

[b60] Kaye G. W. C. & Labay T. H. Tables of Physical and Chemical Constants 5th edn 189, Longman (1986).

[b61] Schweitzer M. H. . A role for iron and oxygen chemistry in preserving soft tissues, cells and molecules from deep time. Proc. R. Soc. B 281, 1775 (2013). 10.1098/rspb.2013.2741 PMC3866414 24285202

[b62] Francis S. E., Sullivan D. J. Jr. & Goldberg D. E. Hemoglobin metabolism in the malaria parasite *Plasmodium falciparum*. Ann. Rev. Microbiol. 51, 97–123 (1997). 9343345 10.1146/annurev.micro.51.1.97

[b63] Susana A. G.-C., Sigifredo A.-G. & Quintin R.-C. Lactoferrin: structure, function and applications. Int. J. Antimicrob. Agents 33, 301.e1–301.e8 (2009). 18842395 10.1016/j.ijantimicag.2008.07.020

[b64] Adlerova L., Bartoskova A. & Faldyna M. Lactoferrin: a review. Vet. Med. 53, 457–468 (2008).

[b65] Parish C. A. . Broad-spectrum antimicrobial activity of hemoglobin. Bioorg. Med. Chem. 9, 377–382 (2001). 10.1016/s0968-0896(00)00263-7 11249130

[b66] Aarden E. M., Burger E. H. & Nijweide P. J. Function of osteocytes in bone. J. Cell Biochem. 55, 287–299 (1994). 7962159 10.1002/jcb.240550304

[b67] Cowin S. C. Mechanosensation and fluid transport in living bone. J. Musculoskel. Neuron Interact. 2, 256–260 (2002). 15758447

[b68] Young M. F. Bone matrix proteins: their function, regulation, and relationship to osteoporosis. Osteoporos. Int. 14, S35–S42 (2003). 12730768 10.1007/s00198-002-1342-7

[b69] Lo Y.-C., Chen C.-I., Chang C.-H., Wang C. & Tsang K.-L. Performance of an infrared beamline for high spatial resolution FTIR microscopy. AIP Conf. Proc. 705, 478–481 (2004).

[b70] Kong J. & Yu S. Fourier transform infrared spectroscopic analysis of protein secondary structures. Acta Biochim. Biophys. Sin. 39, 549–559 (2007). 17687489 10.1111/j.1745-7270.2007.00320.x

