# Supplementary material for: Evidence of preserved collagen in an Early Jurassic sauropodomorph dinosaur revealed by synchrotron FTIR microspectroscopy
Source: Nat Commun. 2017 Jan 31;8:14220. doi: 10.1038/ncomms14220 (PMC5290320; doi:10.1038/ncomms14220)
Supplement: Supplementary Information — Supplementary Figures, Supplementary Tables and Supplementary References [file ncomms14220-s1.pdf]

Supplementary Figures

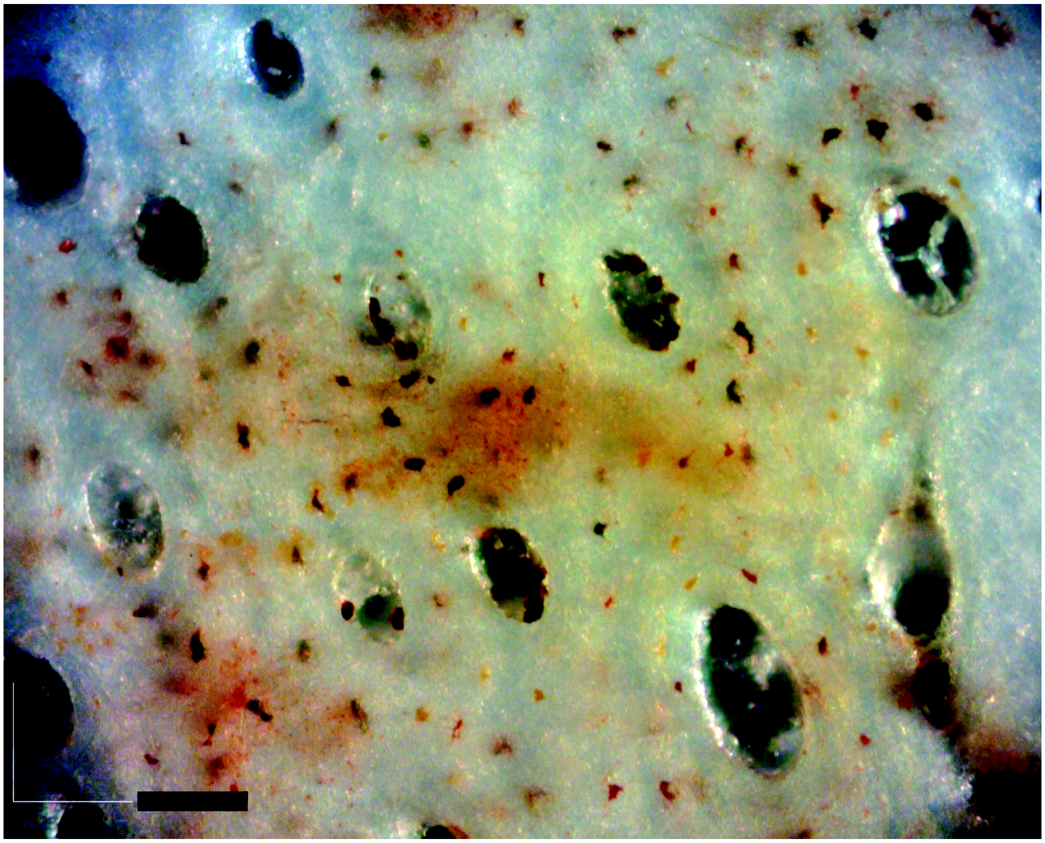

Supplementary Figure 1. Interior structure of *Lufengosaurus* rib in transverse section. Note the presence of the large central vascular canals with hematite particles within the osteons of the compact bone. Lacunae arranged circumferentially within the osteons, with hematite are also visible. Black scale bar equals 50 μm.

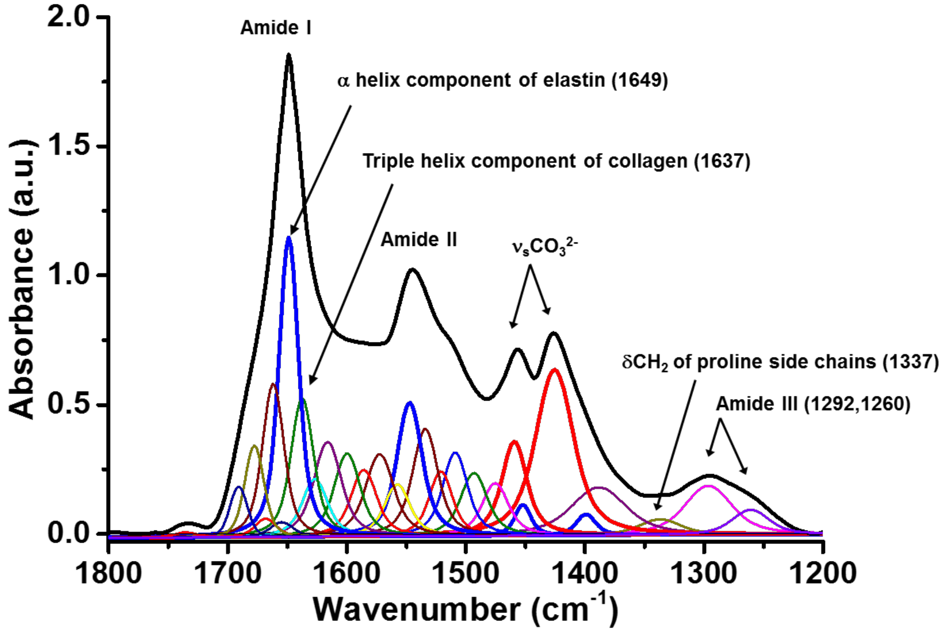

Supplementary Figure 2. Deconvolution curves of the collagen in the *Lufengosaurus* rib. Spectral curve-fitting of SR-FTIR spectrum in the range of 1750-1200 cm^{-1} of the collagen remains within the vascular canals of the adult rib bone of *Lufengosaurus*. The deconvolution of Amide I matched very well with the protein secondary structural peaks (1649 cm^{-1}) and triple helix of collagen I at 1637 cm^{-1}.

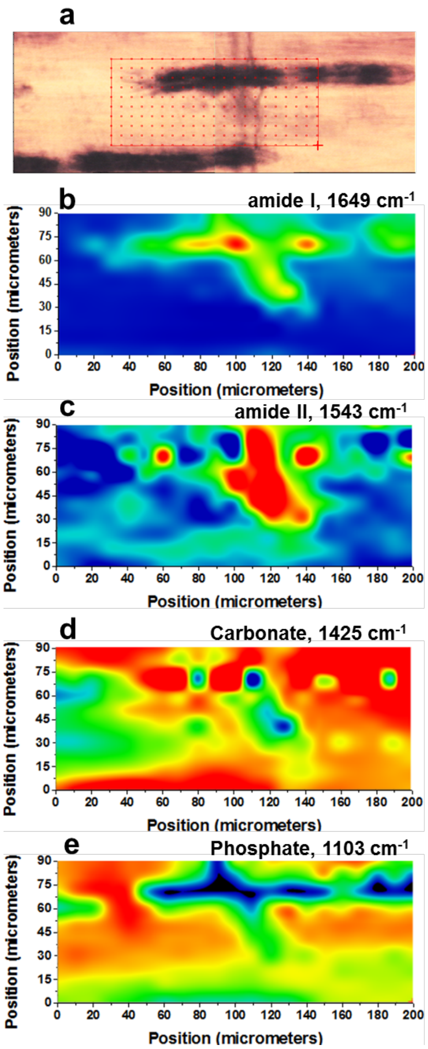

Supplementary Figure 3. Two dimensional optical and FTIR spectral images. (**a**) visible image of preserved collagen infill material of *Lufengosaurus* rib CXPM Z4644. Representative SR-FTIR spectra images of (**b**) Amide I band at 1649 cm^{-1}, (**c**) Amide II band at 1543 cm^{-1}, (**d**) carbonate at 1425 cm^{-1} and (**e**) phosphate at 1103 cm^{-1}.

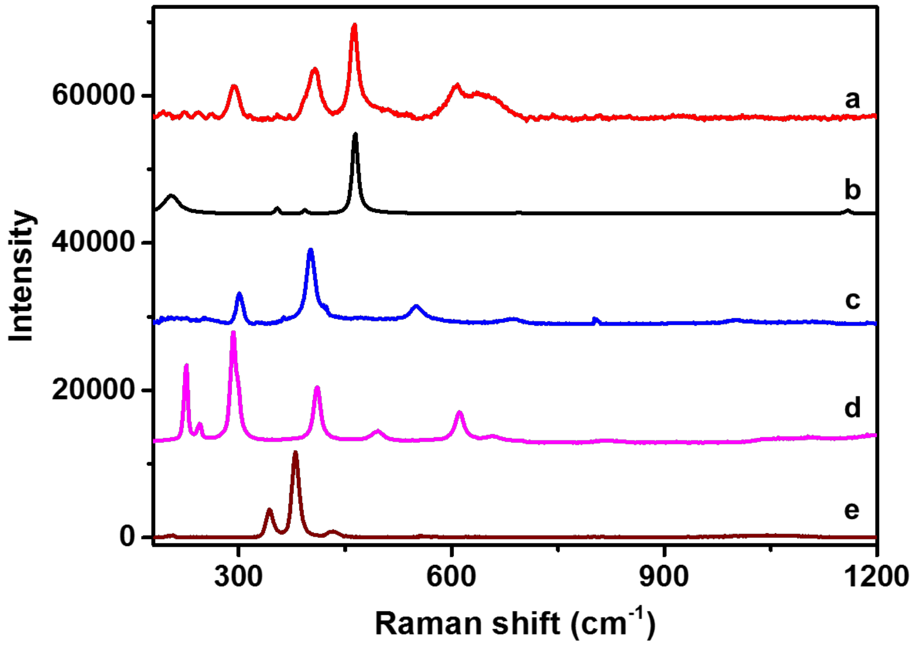

Supplementary Figure 4. Comparative Raman spectra. Red - encasing sediment around the rib fossil; Black - standard quartz; Blue - standard goethite; Pink - standard hematite (α-Fe_2O_3); Brown - standard pyrite (FeS_2) from the RRUFF™ Project (http://rruff.info/Pyrite/R050190).

Supplementary Tables

Supplementary Table 1. FTIR absorption band assignments of fossil bone fossils.

Peak (cm^{-1})	Peak Assignment
3427 (m)	ν_s (OH) stretching vibration of hydroxyl group
3279 (m)	Amide A, ν_s (NH) stretching vibration of amine group of protein
3052 (w)	Amide B, overtone of amide II
2962 (w)	ν_{as} (CH_3), asymmetric stretching vibration of methyl group
2924 (w)	ν_{as} (CH_2), asymmetric stretching vibration of methylene
2872 (w)	ν_s (CH_3), symmetric stretching vibration of methyl group
2851 (w)	ν_s (CH_2), symmetric stretching vibration of methylene
1733 (m)	ν_s (C=O), stretching vibration mode of carbonyl group
1649 (s)	Amide I, ν_s (C=O), stretching vibration of carbonyl group of peptide bond of protein
1637 (m)	Triple helix of collagen
1543 (s)	Amide II, a coupling of ν (C-N) stretching vibration and δ(C-N-H) bending vibration
1454 (s)	$\nu_{3\ or}\ \nu_4$ (C-O-C), bending vibration of CO_3^{2-} group in A and B-type CAP
1424 (s)	ν_1 (C-O), stretching vibration of CO_3^{2-} group in B-type CAP
1337 (w)	δ (CH_2) wagging vibration of proline side chains of type I collagen
1292 (m)	Amide III, a coupling of ν (C-N) stretching vibration and δ (C-N-H) bending vibration, non-polar triple helix of collagen
1260 (m)	Amide III, polar triple helix of collagen and elastin, a coupling of ν (C-N) stretching vibration and δ (C-N-H) bending vibration
1120 (s)	ν (P-O), stretching vibration of PO_4^{3-} group of Mg-doped CAP
1088 (s)	ν_{3a} (P-O), triply degenerate asymmetric stretching vibration of PO_4^{3-} group of CAP
1007 (m)	ν_{3c} (P-O), triply degenerate asymmetric stretching vibration of PO_4^{3-} group of CAP
966 (m)	ν_1 (P-O), triply degenerate asymmetric stretching vibration of PO_4^{3-} group of CAP
870 (w)	ν_2 (P-O), bending vibration of CO_3^{2-} group in CAP

s: strong, m: medium, w: weak

Supplementary Table 2. Comparison of calculated Voigt Amide I band for protein secondary structure.

Present Work Peak/ cm^{-1}	Literature report [1, 2, 5, 6] Peak/ cm^{-1}	Peak Assignment [3, 4, 7]
**1627**	1629	Turns
**1637**	**1637**	Triple helix of collagen
	1633	antiparallel β-sheet
**1649**	1646	α-helix of elastin
**1654**	1652	α-helix (order), elastin
**1661**	1662	α_{11}-helix
**1668**	1668	Turns
**1677**	1676	Random
**1690**	1691	β-sheet

References

1. Wetzel, D. L. Post, G. R. & Lodder. R. A. Synchrotron infrared microspectroscopic analysis of collagens I, III, and elastin on the shoulders of human thin-cap fibroatheromas. *Vib. Spectrosc*. **38**(1–2), 53-59 (2005).

2. Jackson M, Choo L. P., Watson P. H., Halliday W.C.& Mantsch H. H. Beware of connective tissue proteins: assignment and implications of collagen absorptions in infrared spectra of human tissues. *Biochim. Biophys. Acta* **1270** (1), 1-6 (1995).

3. Vedantham, G. Sparks, H. G. Sane, S. U. Tzannis, S. & Przybycien, T. M. A holistic approach for protein secondary structure estimation from infrared spectra in H_2O solutions. *Anal. Biochem.* **285**, 33-49 (2000).

4. Karima B., Razia N., Gilles G & Cyril P. Collagen types analysis and differentiation by FTIR spectroscopy. *Anal. Bioanal. Chem*. **395**(3), 829-37 (2009).

5. Kaye, T. G. Gaugler, G. & Sawlowicz, Z. Dinosaurian soft tissues interpreted as bacterial biofilms. *PLoS ONE* **3**(7), e2808 (2008).

6. Schliephake, H. & Scharnweber, D. Chemical and biological functionalization of titanium for dental implants. *J. Mater. Chem.* **18**, 2404-2414 (2008).

7. Barth, A. & Haris, P. I. Biological and Biomedical Infrared Spectroscopy. IOS Press, Amsterdam, STM Publishing House (2009). ISBN: 978-1-60750-045-2.
